# Enhancing genetic gain through the application of genomic selection in developing irrigated rice for the favorable ecosystem in Bangladesh

**DOI:** 10.3389/fgene.2023.1083221

**Published:** 2023-02-22

**Authors:** Partha S. Biswas, M. M. Emam Ahmed, Wazifa Afrin, Anisar Rahman, A. K. M. Shalahuddin, Rafiqul Islam, Fahamida Akter, Md Abu Syed, Md Ruhul Amin Sarker, K. M. Ifterkharuddaula, Mohammad Rafiqul Islam

**Affiliations:** ^1^ Plant Breeding Division, Bangladesh Rice Research Institute, Gazipur, Bangladesh; ^2^ International Rice Research Institute, IRRI Bangladesh Office, Dhaka, Bangladesh

**Keywords:** genomic selection, genetic gain, breeding cycle, recurrent selection, irrigated rice

## Abstract

Increasing selection differential and decreasing cycle time, the rate of genetic improvement can be accelerated. Creating and capturing higher genetic with higher accuracy within the shortest possible time is the prerequisite for enhancing genetic gain for any trait. Comprehensive yield testing at multi-locations at early generations together with the shortest line fixation time can expedite the rapid recycling of parents in the breeding program through recurrent selection. Genomic selection is efficient in capturing high breeding value individuals taking additive genetic effects of all genes into account with and without extensive field testing, thus reducing breeding cycle time enhances genetic gain. In the Bangladesh Rice Research Institute, GS technology together with the trait-specific marker-assisted selection at the early generation of RGA-derived breeding lines showed a prediction accuracy of 0.454–0.701 with 0.989–2.623 relative efficiency over the four consecutive years of exercise. This study reports that the application of GS together with trait-specific MAS has expedited the yield improvement by 117 kg ha^−1^·year^−1^, which is around seven-fold larger than the baseline annual genetic gain and shortened the breeding cycle by around 1.5 years from the existing 4.5 years.

## Introduction

Rice plays a key role in food security of Bangladesh. Climate change impact and ever-increasing population are pushing tremendous pressure on agriculture for increasing food production. Under the scenario of decreasing arable land annually by 0.43% and diminishing natural resources, no other viable alternative to increase production per unit area (Salam et al., 2020). Rice production in Bangladesh has increased by around four-fold during the last 5 decades through the introduction and use of improved varieties (MVs) and the practice of optimum crop management solutions. Although in recent years Bangladesh has attained self-sufficiency in rice production, it is still not sustainable. Different natural calamities and human-created crises are endangering food security. A study shows that Bangladesh will require 45 million tons of rice in 2050 to feed its 250 million people (Kabir et al., 2015). It will be a great challenge to meet this demand with the current rate of genetic gain in the yield of rice as estimated by Rahman et al. (2022) as 0.24% for winter rice (Boro) and 0.15% for monsoon rice (RLR-T.Aman), respectively. Therefore, the improvement of breeding materials needs to be focused on top of everything. Genetic gain in crops for a particular trait can be enhanced by shortening the breeding cycle, the time span required for the selection of parents from the progenies of a mating between two grandparents, and the recycling of high-value parents in the breeding program. Application of different speed breeding techniques, such as rapid generation advance (RGA), double haploid, embryo rescue, etc. is the effective means of shortening breeding cycle time. Recycling of elite germplasm in the breeding crosses increases the frequency of favorable alleles of quantitative traits like yield. The genomic selection approach expedites the recycling process of parents; can thereby accelerate the rate of genetic gain for yield.

Genomic selection (GS) is a form of marker-assisted selection, which utilizes markers across the entire genome to estimate genomic estimated breeding values (GEBVs) taking additive genetic effects of all genes into account. The GEBVs are directly used for making the selection of individuals for specific trait. As GEBVs can be predicted with or without phenotyping, the selection at early generation is possible, thus reducing breeding cycle time greatly. GS uses a training population of known phenotypes and genotypes to construct a model of each marker’s effect on the trait. The model is then applied to predict the phenotypic performance of the untested individuals having only genotypes. However, the reliability of such predicted phenotype depends on the accuracy of the estimates. The prediction accuracy is estimated from the correlation between the GEBVs of the individuals and measured phenotype for which it is available. The GS has been reported to be more efficient than the phenotypic selection considering resources involvement (Heffner et al., 2009; Jannink et al., 2010; Lorenz et al., 2011; 2012; Rutkoski et al., 2011; Rutkoski et al., 2012; Wang et al., 2012; Onogi et al., 2015; Spindel and Iwata 2018). Since its first application in cattle breeding (Schaeffer, 2006; Hayes et al., 2009; Venot et al., 2016; Wiggans et al., 2017). GS is increasingly being used in both plant and animal breeding programs to accelerate genetic gain of the traits governed by minor genes (Juma et al., 2021). The application of GS in rice was first reported by Grenier et al. (2015) and its use in rice breeding is continuously increasing. The GS in rice has been used for selection against yield (Xu et al., 2014; Grenier et al., 2015; Spindel et al., 2016; Wang et al., 2017), heading date (Onogi et al., 2016), plant height, flowering time (Grenier et al., 2015; Spindel et al., 2016; Wang et al., 2017), panicle weight (Grenier et al., 2015), tiller number, grain number, thousand kernel weight (Xu et al., 2014; Wang et al., 2017), as well as panicle length, secondary branch number, and productive panicle number per plant (Wang et al., 2017). Iwata et al. (2015) suggested that GS could be useful for predicting rice grain shape, with average accuracy ranging from 0.40 to 0.64. The GS accuracies for grain yield ranged approximately from 0.09 to 0.40 across different studies (Xu et al., 2014; Grenier et al., 2015; Spindel et al., 2016; Wang et al., 2017). In a GS study for heading date with 174 backcrossing inbred lines together with its parental lines of rice using different models, Onogi et al. (2016) reported very high accuracy (r > 0.9) across all models. The accuracy for plant height and flowering time ranged approximately from 0.25 to 0.86 in different studies (Grenier et al., 2015; Spindel et al., 2016; Wang et al., 2017). The GS accuracy reported by Xu et al. (2014) for tiller number, grain number, and thousand kernel weight ranged from 0.67 to 0.69 depending on the models used. In general, the GS accuracy in rice studies varies by trait, population, and the models being used. The commonly used genomic prediction models are ridged regression best linear prediction (rrBLUP) (Whittaker et al., 2000; Meuwissen et al., 2001), Bayesian LASSO (BL) (de los Campos et al., 2009; Park and Casella, 2008), reproducing kernel Hilbert spaces (RKHS) (Gianola et al., 2006) regression, and random forest (RF) (Breiman, 2001). The ridge regression best linear unbiased prediction (rrBLUP) model performs adequately well compared to many other models (Spindel et al., 2015; Spindel et al., 2016; Spindel and Iwata 2018). However, prediction accuracy depends on many factors, including the model, crop, size of the reference population, extent of linkage disequilibrium (LD), marker set, and heritability of the trait of interest (Crossa et al., 2010). Accurate phenotyping of a large training population, preferably over multiple environments and years is required to derive accurate predictions due to the interactions between these factors (Rikkerink et al., 2007; Xu and Crouch, 2008; Resende et al., 2012; Desta and Ortiz, 2014). In this paper, we report the progress of testing training populations in multiple environments and further scope of applying GS in enhancing genetic gain in the breeding program aiming to develop rice varieties for the favorable irrigated ecosystem of Bangladesh.

## Materials and methods

Grain yield data of 1445 breeding lines tested in 64 historical trials during 2014–2019 under the irrigated breeding program of Bangladesh Rice Research Institute (BRRI) were used to estimate baseline genetic gain. The trials included only the elite breeding lines and released varieties as standard check varieties with up to a maximum of 8% common entries in the succeeding years. Performance BLUP for yield extracted for each the breeding lines and used to determine baseline gain, while genomic BLUPs for 3767 breeding lines evaluated at multi-locations under 183 trials during 2019–2022 were extracted and used to estimate the rate of changes in genetic improvement of rice yield.

### Extraction of performance BLUP

Two-stage linear mixed model (Piepho et al., 2008; Smith and Cullis 2018) analysis was performed for extracting performance BLUP for the yield of each line. In the first step, each trial was analyzed separately to realize the best linear unbiased estimation (BLUE) following the model:

$$Y_{ij}={\beta X}_{i}+\varepsilon_{ij}$$ (1)

Where, $Y_{ij}$ represents the vector for observed yield for i^th^ observation, β is the fixed effect of i^th^ genotype and $\varepsilon_{ij}$ is the residual error with $\varepsilon_{ij}$ ∼ N(0, $\sigma^{2}\varepsilon$ ) and E $\left(\varepsilon \right.$ ) = 0. The possible blocking factors were modeled to determine which factors led to the lowest Bayesian Information Criterion (Spilke et al., 2010; Piepho et al., 2015). For trials that followed a row-column design, the possible factors were row and column, for those following an RCBD or augmented RCBD, the possible factor was replicated. The R-packages ‘emmeans’ (Lenth et al., 2019) were used to implement the models.

In the second stage, the BLUEs obtained from the first stage model were used as the response variables in the mixed model analysis. The BLUEs for yield within each environment was modeled according to Bates et al. (2015). The model used is as follows:

$$Y_{ij}=\mu +g_{i}+e_{j}+\varepsilon_{ijk}$$ (2)

Where $Y_{ij}$ is the BLUE of each line in environment j, μ is the overall mean, g_i_ is a random effect of line i with g_i_ ∼ N(0, A $\sigma^{2}$ g), where $\sigma^{2}$ g is the genetic variance and A is the additive genetic relationship matrix based on pedigree, $e_{j}$ is a fixed effect of the environment j, $\varepsilon_{ijk}$ is the residual error in k environment with $\varepsilon_{ij}$ ∼ N(0,R $\sigma^{2}\varepsilon$ ), where R is a matrix proportional to the residual error covariance matrix and $\sigma^{2}\varepsilon$ is the error variance. The R-packages lme4 (Bates et al., 2015) were used to implement the models.

### Genotyping and phenotyping of the breeding lines

In total 431, 816, 1491, and 1029 advanced breeding lines of F_7_-F_9_ generations along with five released varieties (BRRI dhan28, BRRI dhan29, BRRI dhan67, BRRI dhan74, and BRRI dhan89, were evaluated for yield at multi-locations during Boro season of 2018–19, 2019–20, 2020–21, and 2021–22, respectively. The trial meta-data can be seen the Supplementary Table S1. Green leaf tissues from a representative plant of each breeding line was collected in labeled glassine bag at 4–5 weeks after transplanting and stored immediately on ice. The samples were stored in a −80°C freezer until processing for genotyping. DNA was isolated and purified according to the modified Cetyltrimmethyl Ammonium Bromide (CTAB) protocol (Aboul-Maaty and Oraby 2019). Genotyping with genome-wide 1024 SNP markers including 92 trait-specific markers named as 1K-RiCA panel (Arbelaez et al., 2019) was performed at an outsourcing genotyping service provider with the help of IRRI Genotyping Services Laboratory, The Philippines. The genotyping data of 1k-RiCA SNPs were filtered using TASSEL v5.0 (Bradbury et al., 2007) following the criteria that the individuals with more than 15% of heterozygous loci were removed, markers with more than 15% of missing values and minor allele frequency below 0.05 were removed. After filtering, 814–889 markers were retained for doing downstream analysis.

### Estimation of genomic estimated breeding values and optimization of training population size

The rrBLUP model was used to estimate the marker effects in R software using mixed. solve function of rrBLUP package (Endelman 2011). Individual GEBVs were then obtained using estimated marker effects. The prediction accuracy from the rrBLUP model was used to estimate GS relative efficiency (REc). Five hundred iterations of cross-validation were used with a random sampling approach, in which 20%, 30%, 40%, 50%, 60%, and 80% of the entries were randomly sampled as training population (TP) for 669 breeding lines tested in the Boro season, 2019-20 to assess the accuracy and optimize TP size for GS. The GS accuracy was estimated as the correlation coefficient of the GEBVs and the phenotypic values for all accessions. The average accuracy realized from the random sampling was reported as the mean correlation coefficient values from 500 runs. The REc was estimated using the equation:

$${RE}_{c}=\frac{r_{G.O}}{\sqrt{H^{2}}}$$ (3)

Where $r_{G.O}$ is the accuracy of GS and H^2^ is the estimated heritability

### Sparse testing of training population

**FIGURE 1:**
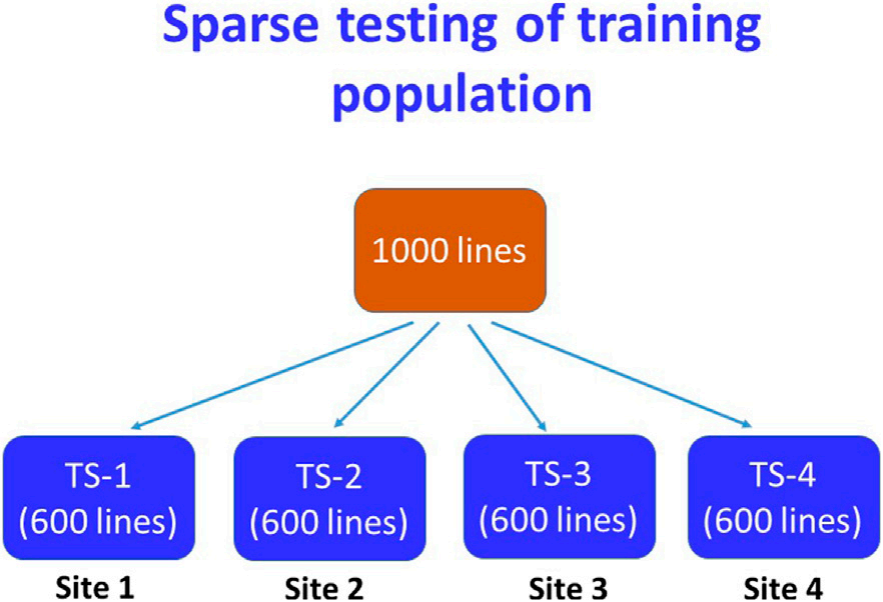
A scheme of sparse testing of training population for the genomic selection followed for the irrigated ecosystem. In this scheme, 40% of lines of the breeding population are common in all four TPs and the remaining lines were sampled by 25% at each time avoiding duplication among the TPs.

The efficiency of GS depends on the relative proportion (size) and genetic relationship of the training population with the whole breeding population under the model. Based on the accuracy of prediction with the 500-fold cross-validation of varying sizes training population, four training populations comprising 60% of the total breeding lines were considered for yield testing at four locations following the sparse testing model of GS (Jarquin et al., 2020; Atanda et al., 2021; Atanda et al., 2022). An example scheme of sparse testing of TP has been shown in Figure 1. To save resources and to make connectivity between the trials, 40% of the total entries of the whole breeding population were sampled first as a common share to each training population. The common share of the TP was constructed in such a way that it contained the breeding lines of all the crosses in the study with at least one parent common. The remaining portion of the TP was sampled randomly from the remaining lines of the breeding population taking 25% lines at each time without replacement to avoid resampling of the same entry in the next round of sampling.

### Estimation of genetic gain for yield

Genetic gain was estimated as the rate of change in breeding value per unit of time following the procedure reviewed by Garrick (2010). Briefly, performance BLUPs of 1108 individual lines tested in 44 trials during 2016–2019 were extracted by using Eq 1, 2 in the two-stage linear mixed model described above. These BLUP values were regressed on the year when the lines were evaluated to get the baseline genetic gain. Genomic BLUP values of each line were extracted from the trials conducted during 2020, 2021, and 2022 following the same principle using R-package rrBLUP, and the rate change in genetic improvement in yield was determined by regressing on the trial year. The regression line was fitted on the scatter plots at 95% confidence intervals following the formula given below,

$$CI=\bar{x}\;\pm \;z^{*}\times \frac{\sigma }{\sqrt{n}}$$ (4)

where, $\bar{x}$ is the sample mean, $z^{*}$ is the level of confidence, *σ* is the population standard deviation and *n* is the sample size

The regression co-efficient i.e. genetic gain was subjected to *t*-test for level of significance.

### Post facto analysis of BRRI crosses

All the crosses made for the irrigated breeding program during 1994–2022 were retrieved from the BRRI crossing database. The initial filtering boundary to the year “1994” was set taking the released year of BRRI dhan28 and BRRI dhan29 into consideration. BRRI dhan28 and BRRI dhan29 are the widely cultivated varieties in the irrigated ecosystem of Bangladesh. The frequency of the crosses using these two varieties as parents were estimated in the percentage of the total number of crosses made under the irrigated breeding program after their release.

## Results

### Assessment of baseline genetic gain for yield

**FIGURE 2:**
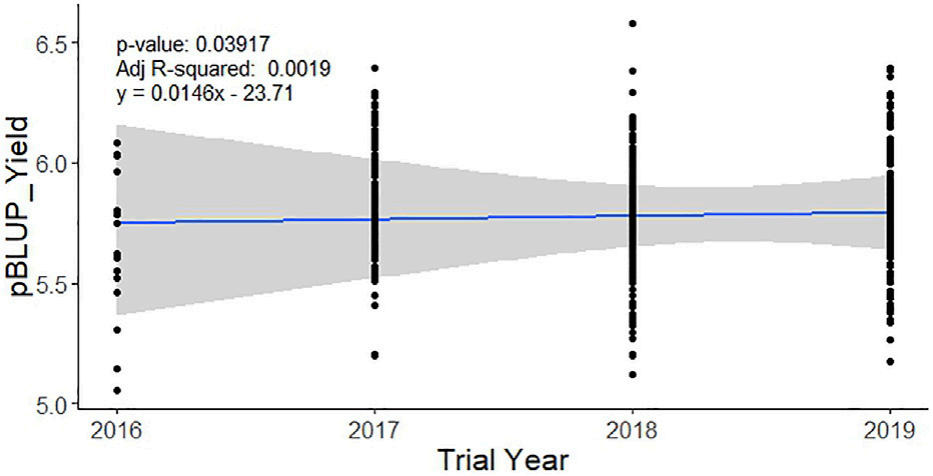
Baseline genetic gain for yield in the irrigated breeding program of BRRI during 2016–2019. Performance BLUP for the yield of each line was regressed over the year of the trial to extract the rate of genetic improvement per year.

The analysis of 1108 individual lines tested in 44 trials from 2016 to 2019 under irrigated favorable ecosystem showed the yield BLUP varied from 5.79 t ha^−1^ (in 2016) to 5.88 t ha^−1^ (in 2019) with an average of 5.78 t ha^−1^. The variation among the tested lines was much narrow (up to 4.65%) across the years (Table 1). Importantly, trial size (no. of entries), locations, and design were variable across the year. The simple regression analysis of the BLUP values with the trial year showed a baseline genetic grain for the yield of 0.0174 t ha^−1^·year^−1^ (Figure 2).

**TABLE 1:** Meta data and descriptive statistics of the analysis for the yield of 1108 individual breeding lines/varieties tested in 44 trials during Boro season of 2016–2019 under favorable ecosystems.

	Year of field trial				
	2016	2017	2018	2019	Across the year
No. of genotypes tested	63	324	290	431	1108
No of trial	10	4	4	26	44
No. of location	1	7	4	19	31
Trial design	RCB	RCB, ARCB	RCB, RC	RCB, RC	—
Range (t ha^−1^)	5.05–6.15	5.11–6.56	5.12–6.58	5.11–6.56	—
Average (t ha^−1^)	5.79 ± 0.20	5.77 ± 0.27	5.82 ± 0.26	5.88 ± 0.27	—
CV (%)	3.46	4.65	4.46	4.45	—

RCB, randomized complete block; SA, systematic arrangement; ARCB, augmented randomized complete block; RC, Row-Column.

### Assessment of the accuracy of genomic selection

**FIGURE 3:**
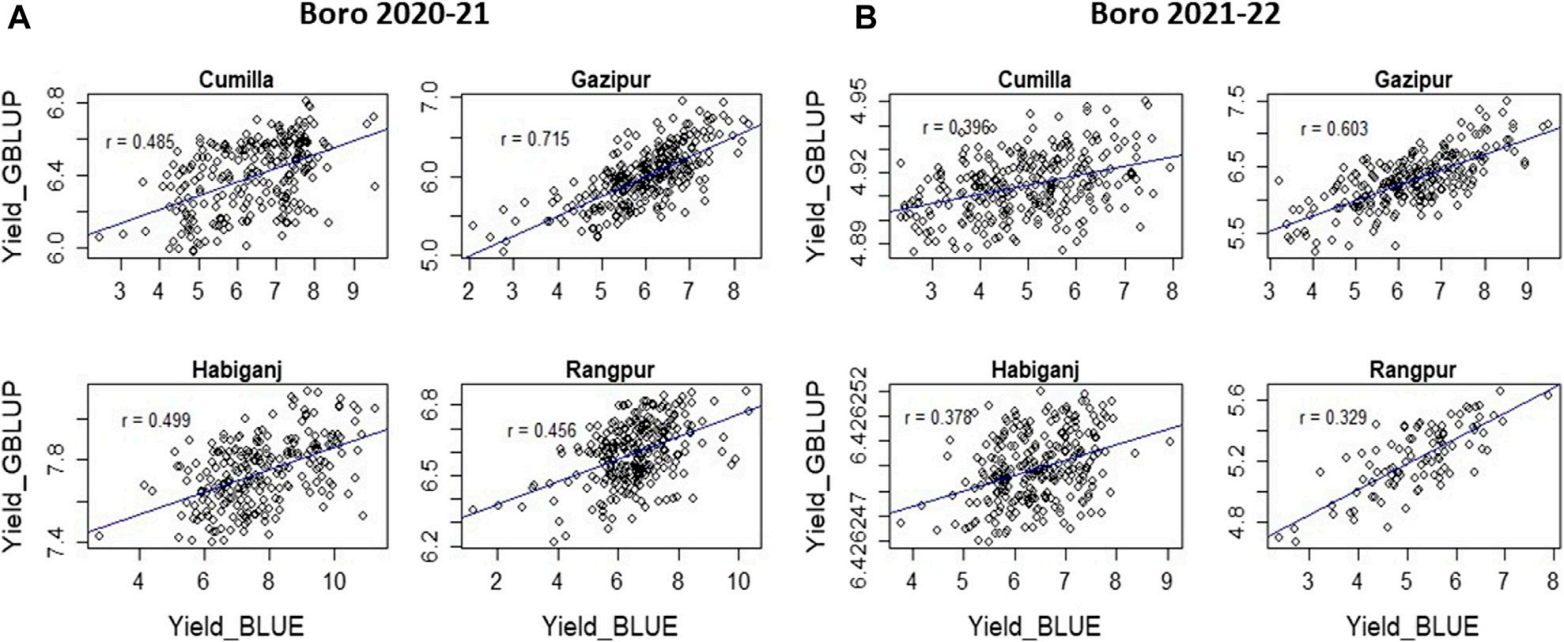
Accuracy of genomic prediction estimated from OYT trial conducted with 799 lines during Boro 2020–21 **(A)** and OYT trial conducted with 548 lines during Boro 2021–22 **(B)** at four locations in Bangladesh.

**FIGURE 4:**
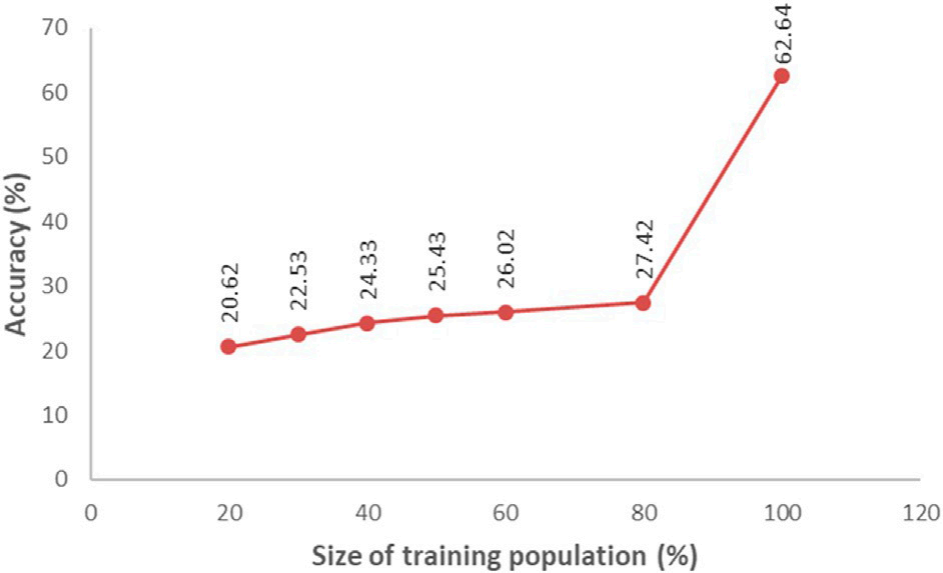
Mean prediction accuracy for yield in relation to training population size determined from a population of 669 breeding lines tested in Boro season at multi-locations during 2020. Average accuracy was determined from 500 iterations of cross-validation of different sizes (proportion of the breeding population) of the training population.

Accuracy of GS was estimated through Pearson’s correlation between the predicted performance and the actual performance. The GS accuracy in an observational yield trial (OYT) trial with 799 breeding lines conducted during Boro season 2020–21 at four locations (Cumilla, Gazipur, Habiganj, and Rangpur) following sparse testing model ranged from 0.456 to 0.715 (Figure 3), while in the trials of Boro 2021–22 season, it varied from 0.396 to 0.757 across the sites with a different set of 618 breeding lines. On the other hand, the GS accuracy in multiple trials conducted at different regional research stations with different sets of breeding lines in Boro 2018–19 (431 lines in 26 trials), Boro 2019–20 (816 lines in 43 trials), Boro 2020-21 (1491 lines in 71 trials), Boro 2021–22 (1029 lines in 43 trials) were 0.672, 0.701, 0.454, and 0.509, respectively (Table 2; Supplementary Figure S1). A study aiming to optimize training population size with 669 breeding lines tested in the Boro season of 2020 at multi-locations showed that average prediction accuracy gradually increased up to 27.42% with the increase of training population size (up to when 80% of the entries of the breeding population was included in the training population) and afterward it sharply jumped to 62.64% when 100% lines were in the training population (Figure 4).

**TABLE 2:** Prediction accuracy and relative efficiency of prediction in selecting high breeding value parents during four consecutive Boro seasons, 2018–19 to 2021–22.

Season	No. of lines tested (in different trials)	No. of parents selected based on GEBV for yield	Prediction accuracy	Relative efficiency of prediction
Boro 2018-19	431 (26)	22	0.672	1.933
Boro 2019-20	816 (43)	23	0.701	2.623
Boro 2020-21	1491 (71)	31	0.454	0.989
Boro 2021-22	1029 (43)	25	0.509	1.580

### Genomic selection for choosing parents

The GS approach has been in routine use since Boro 2018–19 season for selecting high breeding value lines to recycle in the crossing program. In Boro 2018–19, 27 parents out of 431 lines tested in different classes of trials at 19 locations across the country were selected based on GEBV for yield (Table 2). Similarly, 23 parents out of 816 lines tested during Boro 2019–20, 31 parents out of 1491 lines tested during Boro 2020–21, and 25 parents out of 1029 lines tested during Boro 2021–22 were selected based on GEBV for yield and used in the crossing program. The prediction accuracy and relative prediction efficiency varied from 0.454 to 0.701 and 0.989 to 2.623, respectively. The Supplementary Figure S1 shows the association of GEBV for yield and the BLUE for yield.

### Genomic selection at the early generation yield testing stage

The sparse testing model of GS allows the evaluation of a large set of lines under different sets of training populations at multi-locations. This method was practiced in the irrigated breeding program at the OYT stage, which is the first stage of yield trial. In the 2020–21 Boro season, out of 650 breeding lines, 249 lines at Cumilla, 289 lines at Gazipur, 232 lines at Habiganj, and 275 lines at Rangpur were tested as training population. The genomic prediction of these four sites showed a range of predicted yield between 5.94–6.81 t/ha at Cumilla, 5.04–6.96 t/ha at Gazipur, 7.40–8.18 t/ha at Habiganj, and 6.22–6.86 t/ha at Rangpur. The prediction accuracy with the training population was 0.456 at Cumilla, 0.715 at Gazipur, 0.499 at Habiganj, and 0.456 at Rangpur (Figure 3). On the other hand, out of 548 breeding lines, 292 lines at Cumilla, 249 lines at Gazipur, 280 lines at Habiganj and 125 lines at Rangpur tested as training population in Boro 2021–22 showed prediction accuracy 0.396, 0.603, 0.378, and 0.329, respectively. The predicted yield based on GEBV was found 4.89–4.95 t/ha at Cumilla, 5.23–7.50 t/ha at Gazipur, 6.4262–6.4263 t/ha at Habiganj, and 4.70–5.66 t/ha at Rangpur.

### Genomic selection at F_5:6_ LST without yield evaluation

**FIGURE 5:**
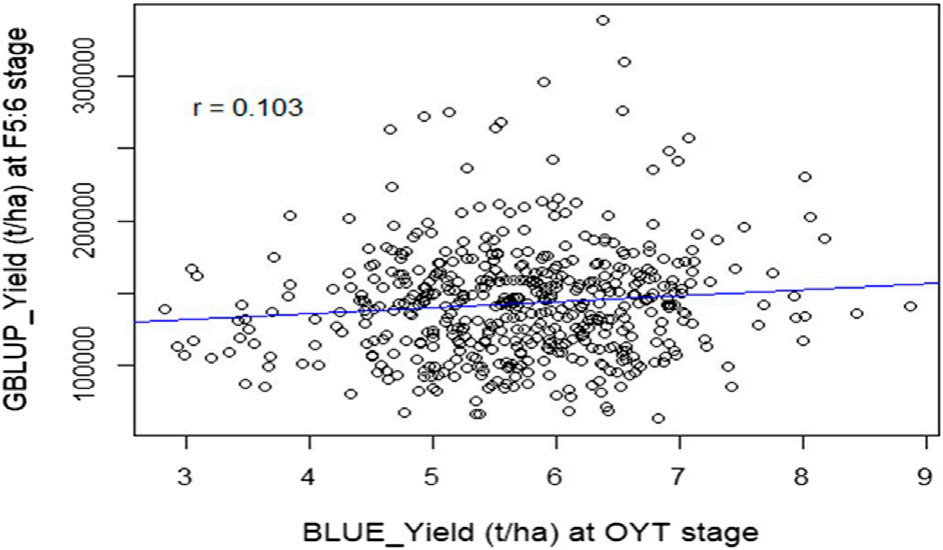
Scatter plot of gBLUP for the yield of F_5:6_ lines against the corresponding BLUE values estimated from yield testing in OYT during the Boro season of 2022. The value “r” indicates the accuracy of the prediction.

GS was performed on 505 F_5:6_ LST (Line Stage Testing) lines derived from 77 crosses using genotyping data of 860 SNPs and yield data of their 39 parents. The prediction accuracy was found 0.103 when correlation analysis was performed between the predicted yield (gBLUP) of the LST lines and the BLUE values extracted for the same set of lines from the OYT trials in the Boro season of 2021–22 (Figure 5). However, the correlation coefficient between the gBLUP and BLUEs of the parents was as high as 0.708.

### Estimation of current genetic gain

**FIGURE 6:**
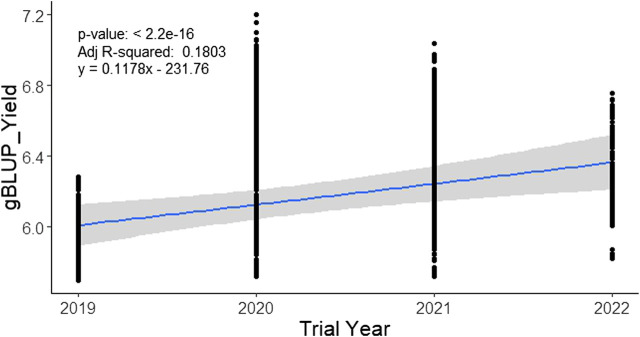
Genetic gain for yield in the irrigated breeding program of BRRI during 2019–2022. Genomic BLUP for the yield of each line was regressed over the year of the trial to obtain the rate of genetic improvement per year.

The analysis of 3767 individual lines (with a maximum of 8.4% duplicates over the year) tested from 2019 to 2022 under irrigated favorable ecosystem showed a range of gBLUP for yield (5.69–7.2 t/ha) over the trial year (Table 3). In 2019, it varied from 5.69 t/ha to 6.28 t/ha with an average of 5.90 t/ha, in 2020, it was 5.72 t/ha to 7.2 t/ha with an average of 6.42 t/ha. In total, 1491 breeding lines were evaluated in 2021 and gBLUP varied from 5.72 t/ha to 7.03 t/ha. In 2022, 1029 lines were evaluated under 43 trials at 27 locations. The gBLUP extracted for each line in this year varied from 5.82 t/ha to 6.76 t/ha with an average value of 6.16 t/ha. The variation among the tested lines in gBLUP was a maximum of up to 5.61% across the years. The regression analysis of the gBLUP with the trial year showed a change in rate of genetic improvement in yield by 0.1178 t ha^−1^ year^−1^ (Figure 6).

**TABLE 3:** Genomic BLUP for yield extracted from the breeding trials conducted at different locations during 2019–2022 under irrigated favorable ecosystem in Boro season.

	Year of field trial				
	2019	2020	2021	2022	Across the year
No. of genotypes tested	431	816	1491	1029	3767
No of trial (location)	26 (19)	43 (21)	71 (23)	43 (27)	183 (90)
Range (t ha^−1^)	5.69–6.28	5.72–7.20	5.72–7.03	5.82–6.76	5.69–7.20
Average (t ha^−1^)	5.90 ± 0.18	6.42 ± 0.06	6.37 ± 0.24	6.16 ± 0.14	—
CV (%)	3.12	5.61	3.81	2.27	—

## Discussion

Genetic gain is the amount of increased genetic improvement of a population over time due to intervention of selection for specific traits. It is usually estimated per unit of time and/or per unit area and/or per unit investment. Measuring the genetic gain of rice breeding programs is extremely important, as it is the staple food crop in Bangladesh. The analysis of baseline genetic grain for yield based on trial year shows that the irrigated breeding program of Bangladesh Rice Research Institute had a value of 0.0146 t ha^−1^ year^−1^ (Figure 1) from 2016 to 2019. This rate of genetic improvement is quite low and inadequate compared to the expected genetic gain of at least 0.044 t ha^−1^ per year (approximately 1% annually) (Kabir et al., 2015) to meet Bangladesh’s requirements through 2050 for ensuring food security. In a study using historical series data of the released varieties over 50 years from 1970 to 2020, Rahman et al. (2022) reported the baseline genetic gain as 0.01 t ha^−1^ year^−1^ for both rainfed lowland (monsoon rice) and irrigated rice (winter rice). However, these rates are consistent with those observed for other South Asian rice breeding programs serving favorable environments (Kumar et al., 2021). In general, low rates of genetic gain in South and Southeast Asian rice breeding are likely due to long breeding cycles caused by repeated use of older, popular varieties as parents, and by limited selection intensity for yield in multi-location trials. Post facto analysis of BRRI crosses showed that in its irrigated breeding program, the most popular varieties BRRI dhan28 and BRRI dhan29 were repeatedly used as parents. Also, frequent use of landrace varieties in the crossing programs (Supplementary Table S2) without proper pre-breeding activities has resulted in limited improvement in additive breeding value for grain yield of rice in the irrigated breeding program.

Breeder’s equation (Lush 1937) suggests that by increasing the selection differential per unit of time or cost, the genetic gain can be enhanced. Increased selection differential depends on the trial heritability/accuracy, selection intensity, the genetic variance of the trait, and re-cycling time or length of the breeding cycle. Multi-location trials improve trial heritability. The inclusion of high-breeding-value parents in the breeding program increases genetic variance and selection intensity. Cutting-edge speed breeding techniques, such as RGA, double haploid, embryo rescue, etc. have shown promise in reducing breeding cycle time (Cobb et al., 2019; Ahmar et al., 2020; Shanmugavel et al., 2022). GS provides an opportunity to hasten the cycle of selection. It also showed the potential to select high-breeding value individuals from early-generation populations without extensive field testing. GS has been shown effective in wheat (Bonnett et al., 2022), maize (Beyene et al., 2021), barley (Sallam et al., 2015), and even rice (Xu et al., 2021). In our study, we also found that genetic gain per unit of time is much faster in the GS strategy than in the conventional selection methods. Since 2018-19, the GS approach is routinely practiced in selecting high-breeding value parents for recycling in the breeding program. One hundred Six superior lines with high GEBVs comprising 27 lines from the breeding trials conducted in 2018–19, 23 lines from 2019–20’s trial 31 from 2020–21’s trial and 25 from 2021–22’s trial were isolated and recycled in the crossing program (Tables 2, 3) and thereby, frequency of favourable alleles for yield has been increased in the breeding population. GS strategy helped grab the high GEBV lines as it accounts for the marker effect with the phenotypic performance (Contaldi et al., 2021).

Prediction accuracy is a very important factor for applying GS in filtering selection candidates. The prediction accuracy depends on various factors including the model used in the GS scheme. The rrBLUP is the frequently used GS model in the field of plant breeding. However, Rutkosky et al. (2012) reported Reproducing Kernel Hilbert Spaces (RHKS) regression and Random Forest (RF) regression as the most accurate models for genomic prediction. The simplicity of rrBLUP to extract marker effect made it popular among plant breeders. Thus, we used this model in our study for genomic prediction of the untested breeding lines. Another factor is the size of the training population which significantly affects the accuracy of genomic prediction. In a study of optimization of the training population size, we found that prediction accuracy gradually increases with the increase of breeding lines and sharply increases when TP contains more than 80% of the breeding lines of a validation population (Figure 4). Data quality of the training population is another important factor for GS accuracy. The heritability of a trial is an ideal indicator of data quality. Heritability for a quantitative trait like yield between 0.4 to 0.6 is considered to be optimum for the best quality data.

The GS technology not only can capture high-value parents but can be used to predict the performances of the untested (validation population) lines together with the tested lines, thus it saves resources required for the field testing of the whole population. The sparse testing approach of GS (Jarquin et al., 2020), in which the breeding population is subsetted into training populations with different but genetically related lines by pedigree for field testing, saves resources further by reducing duplication of the lines across the locations. Applying the sparse testing model, we evaluated 650 breeding lines in 2020–21 and 548 breeding lines in 2021–22 at four locations without testing all of them in all locations. The level of prediction accuracy observed at Cumilla (0.456 and 0.396), Gazipur (0.715 and 0.603), Habiganj (0.499 and 0.378) and Rangpur (0.456 and 0.329) during 2020–21 and 2021–22 (Figure 3), respectively suggests the reliability of the predicted performance of the untested breeding lines**.** The GS accuracy for grain yield of rice was reported with a range from 0.09 to 0.40 in many studies conducted by Xu et al. (2014), Grenier et al. (2015), Spindel et al. (2016), Wang et al. (2017). In addition, GS accuracy in the multiple advanced yield trials conducted at different regional research stations with different sets of breeding lines in Boro 2018–19 (431 lines 26 trials), Boro 2019–20 (816 lines in 43 trials), Boro 2020–21 (1491 lines in 71 trials), Boro 2021–22 (1029 lines in 43 trials) season were 0.672, 0.701, 0.454, and 0.509 with high (1.933, 2.623, 0.989, and 1.58, respectively) relative efficiency of prediction (Table 2; Supplementary Figure S1). These results indicated that the sparse testing model of GS was as effective for capturing expected selection candidates as the GS models with the same set of training population tested across the sites.

**FIGURE 7:**
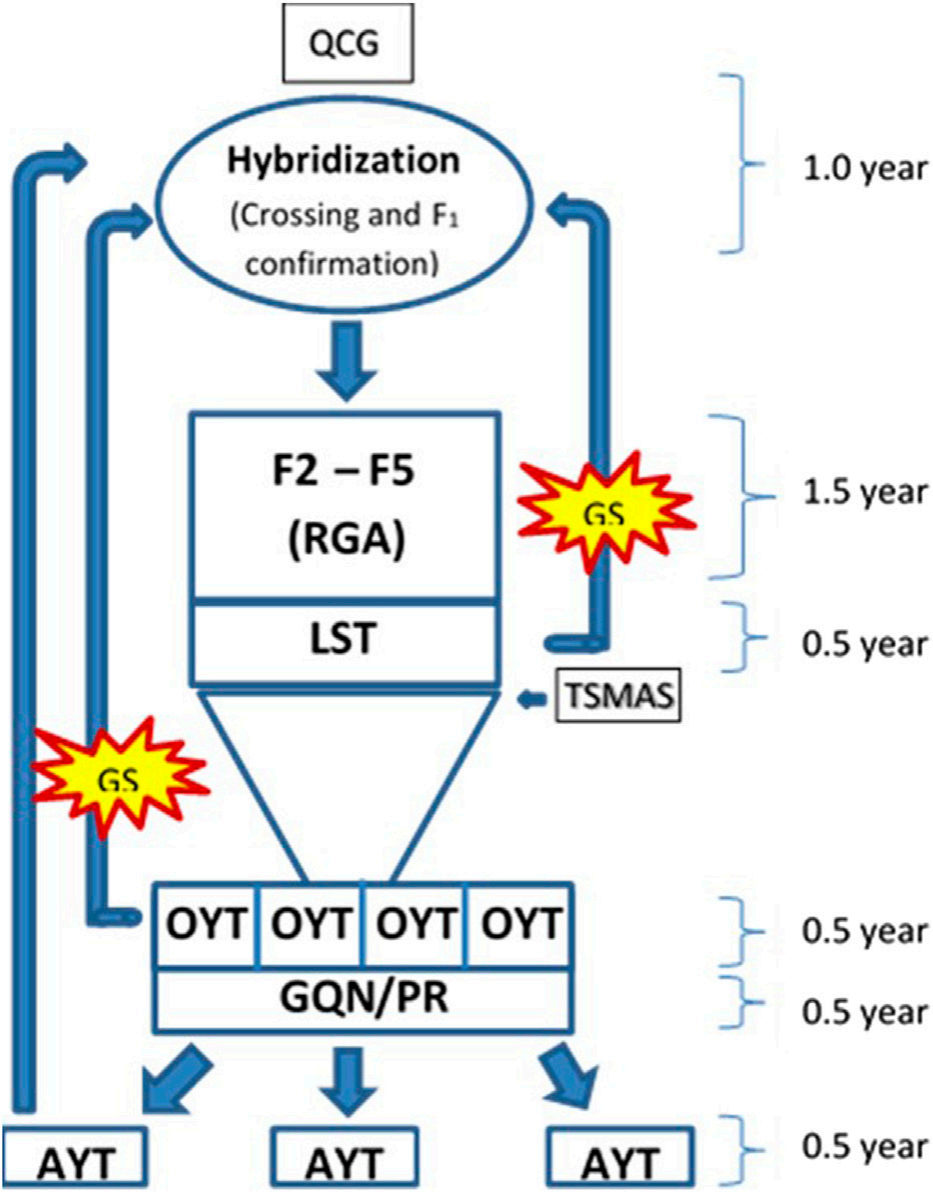
Schematic diagram of core part of breeding pipeline of irrigated rice breeding program for favorable ecosystem showing a breeding cycle of 4.5 years. QCG, Quality control genotyping used for parental purification and hybridity test of crosses. LST, line stage testing, is used for genotyping with trait specific markers and seed multiplication of the selected entries. OYT, observational yield trial, is the initial multi-location yield trial, AYT, advanced yield trial, is performed at multi-location with the selected entries from OYT. GQN, grain quality and nutrition, and PR, pest reaction of the OYT lines are checked before promotion to AYT.

The rate of genetic gain can be improved by increasing selection differential with sufficient accuracy and decreasing cycle time (Cobb et al., 2019). Before adopting the RGA technique in advancing segregating populations, the breeding cycle length was roughly 8–10 years in the breeding program of BRRI and IRRI (Collard et al., 2017; Cobb et al., 2019). The cycle time of BRRI’s breeding programs has been cut down from 8–10 years to 4–5 years by the use of RGA techniques (Rahman et al., 2019) as shown in Supplementary Table S3. For further reduction in cycle time, in this study, the GS technique was used for predicting the performance without yield testing of a portion of the total breeding lines at the initial yield trial called OYT and found reliable prediction accuracy in the trials of Boro 2020–21 and Boro 2021–22 (Figure 3). Also, marker-assisted selection was performed for different target traits viz. cold tolerance, disease and insect resistance, grain quality, etc. using trait makers embedded within the 1K-RiCA panel for filtering the superior selection candidates. Thus, GS has cut down another 0.5–1.0 years that would be required for yield testing in the advanced yield trials and phenotyping for grain quality and pest reaction before selecting parents for recycling (Figure 7). Applying GS together with MAS for key target traits at the line fixation stage (F_4_-F_5_) has further reduced cycle time by at least half a year and thereby increased the rate of genetic gain as indicated in Figure 6. However, the accuracy of prediction was compromised greatly (Figure 5). Careful selection of breeding lines in the training population, recycling only the elite lines with adequate genetic variance for the traits as parents in the crossing program and good quality phenotyping data could improve prediction accuracy. Non-etheless, practicing GS for the consecutive 4 years from 2019 to 2022, genetic improvement for yield has been recorded at the rate of 117 kg ha^−1^ year^−1^, which is around 6.77 fold higher than the baseline gain.

Based on the above findings it can be concluded that by applying GS, superior lines with high breeding value can be reliably captured with and without extensive field phenotyping. GS approach particularly sparse testing of the training population saved resources required for the phenotyping without sacrificing prediction accuracy. Moreover, results show that by practicing GS at OYT level, breeding cycle time could be reduced to 3.5 years from the existing 4.5 years. If GS is performed at the LST stage, cycle time can be further reduced by another half a year.

## Data availabilty statement

The original contributions presented in the study are publicly available. This data can be found here: https://www.ebi.ac.uk/eva/?eva-study=PRJEB59909.

## References

[B1] Aboul-Maaty N. A. F. Oraby H. A. S. (2019). Extraction of high-quality genomic DNA from different plant orders applying a modified CTAB-based method. Bull. Natl. Res. Cent. 43, 25. 10.1186/s42269-019-0066-1

[B2] Ahmar S. Gill R. A. Jung K. H. Faheem A. Qasim M. U. Mubeen M. (2020). Conventional and molecular techniques from simple breeding to speed breeding in crop plants: Recent advances and future outlook. Int. J. Mol. Sci. 21, 2590. 10.3390/ijms21072590 32276445 PMC7177917

[B3] Arbelaez J. D. Dwiyanti M. S. Tandayu E. Llantada K. Jarana A. Ignacio J. C. (2019). 1k-RiCA (1K-Rice Custom Amplicon) a novel genotyping amplicon-based SNP assay for genetics and breeding applications in rice. Rice 12, 55. 10.1186/s12284-019-0311-0 31350673 PMC6660535

[B4] Atanda S. A. Olsen M. Crossa J. Burgueño J. Rincent R. Dzidzienyo D. (2021). Scalable sparse testing genomic selection strategy for early yield testing stage. Front. Plant Sci. 12, 658978. 10.3389/fpls.2021.658978 34239521 PMC8259603

[B5] Atanda S. A. Govindan V. Singh R. Robbins K. R. Crossa J. Bentley A. R. (2022). Sparse testing using genomic prediction improves selection for breeding targets in elite spring wheat. Theor. Appl. Genet. 135, 1939–1950. 10.1007/s00122-022-04085-0 35348821 PMC9205816

[B6] Bates D. Mächler M. Bolker B. Walker S. (2015). Fitting linear mixed-effects models using lme4. J. Stat. Softw. 67, 1–48. 10.18637/jss.v067.i01

[B7] Beyene Y. Gowda M. Pérez-Rodríguez P. Olsen M. Robbins K. R. Burgueño J. (2021). Application of genomic selection at the early stage of breeding pipeline in tropical maize. Front. Plant Sci. 12, 685488. 10.3389/fpls.2021.685488 34262585 PMC8274566

[B8] Bonnett D. Li Y. Crossa J. Dreisigacker S. Basnet B. Pérez-Rodríguez P. (2022). Response to early generation genomic selection for yield in wheat. Front. Plant Sci. 12, 718611. 10.3389/fpls.2021.718611 35087542 PMC8787636

[B9] Bradbury P. J. Zhang Z. Kroon D. E. Casstevens T. M. Ramdoss Y. Buckler E. S. (2007). TASSEL: Software for association mapping of complex traits in diverse samples. Bioinformatics 23, 2633–2635. 10.1093/bioinformatics/btm308 17586829

[B10] Breiman L. (2001). Random forests. Mach. Learn. 45, 5–32. 10.1023/A:1010933404324

[B11] Cobb J. N. Juma R. U. Biswas P. S. Arbelaez J. D. Rutkoski J. Atlin G. (2019). Enhancing the rate of genetic gain in public-sector plant breeding programs: lessons from the breeder’s equation. Theor. Appl. Genet. 132, 627–645. 10.1007/s00122-019-03317-0 30824972 PMC6439161

[B12] Collard B. C. Beredo J. C. Lenaerts B. Mendoza R. Santelices R. Lopena V. (2017). Revisiting rice breeding methods–evaluating the use of rapid generation advance (RGA) for routine rice breeding. Plant Prod. Sci. 20, 337–352. 10.1080/1343943X.2017.1391705

[B13] Contaldi F. Cappetta E. Esposito S. (2021). “Practical workflow from high-throughput genotyping to genomic estimated breeding values (GEBVs),” in Crop breeding. Methods in molecular biology. Editor Tripodi P. (New York, NY: Humana), 2264. 10.1007/978-1-0716-1201-9_9 33263907

[B14] Crossa J. de los Campos G. Pérez P. Gianola D. Burgueño J. Araus J. L. (2010). Prediction of genetic values of quantitative traits in plant breeding using pedigree and molecular markers. Genetics 186, 713–724. 10.1534/genetics.110.118521 20813882 PMC2954475

[B15] de los Campos G. Naya H. Gianola D. Crossa J. Legarra A. Manfredi E. (2009). Predicting quantitative traits with regression models for dense molecular markers and pedigree. Genetics 182, 375–385. 10.1534/genetics.109.101501 19293140 PMC2674834

[B16] Desta Z. A. Ortiz R. (2014). Genomic selection: genome-wide prediction in plant improvement. Trends Plant Sci. 19, 592–601. 10.1016/j.tplants.2014.05.006 24970707

[B17] Endelman J. B. (2011). Ridge regression and other kernels for genomic selection with R package rrBLUP. Plant Genome 4, 250–255. 10.3835/plantgenome2011.08.0024

[B18] Garrick D. J. (2010). An animal breeding approach to the estimation of genetic and environmental trends from field populations. J. Anim. Sci. 88, E3–E10. 10.2527/jas.2009-2329 19820056

[B19] Gianola D. Fernando R. L. Stella A. (2006). Genomic-assisted prediction of genetic value with semiparametric procedures. Genetics 173, 1761–1776. 10.1534/genetics.105.049510 16648593 PMC1526664

[B20] Grenier C. Cao T. V. Ospina Y. Quintero C. Châtel M. H. Tohme J. (2015). Accuracy of genomic selection in a rice synthetic population developed for recurrent selection breeding. PLoS One 10, e0136594. 10.1371/journal.pone.0136594 26313446 PMC4551487

[B21] Hayes B. J. Bowman P. J. Chamberlain A. J. Goddard M. E. (2009). Invited review: genomic selection in dairy cattle: progress and challenges. J. Dairy Sci. 92, 433–443. 10.3168/jds.2008-1646 19164653

[B22] Heffner E. L. Sorrells M. E. Jannink J. L. (2009). Genomic selection for crop improvement. Crop Sci. 49, 1–12. 10.2135/cropsci2008.08.0512

[B23] Iwata H. Ebana K. Uga Y. Hayashi T. (2015). Genomic prediction of biological shape: elliptic fourier analysis and kernel partial least squares (PLS) regression applied to grain shape prediction in rice (oryza sativa L.) PLoS One 10, e0120610. 10.1371/journal.pone.0120610 25825876 PMC4380318

[B24] Jannink J. L. Lorenz A. J. Iwata H. (2010). Genomic selection in plant breeding: from theory to practice. Briefings Funct. Genomics 9, 166–177. 10.1093/bfgp/elq001 20156985

[B25] Jarquin D. Howard R. Crossa J. Beyene Y. Gowda M. Martini J. W. (2020). Genomic prediction enhanced sparse testing for multi-environment trials. G3 Genes, Genomes, Genet. 10, 2725–2739. 10.1534/g3.120.401349 PMC7407457 32527748

[B26] Juma R. U. Bartholomé J. Thathapalli Prakash P. Hussain W. Platten J. D. Lopena V. (2021). Identification of an elite core panel as a key breeding resource to accelerate the rate of genetic improvement for irrigated rice. Rice 14, 92–22. 10.1186/s12284-021-00533-5 34773509 PMC8590642

[B27] Kabir M. S. Salam M. U. Chowdhury A. Rahman N. M. F. Iftekharuddaula K. M. Rahman M. (2015). Rice vision for Bangladesh: 2050 and beyond. Bangladesh Rice J. 19, 1–18. 10.3329/brj.v19i2.28160

[B28] Kumar A. Raman A. Yadav S. Verulkar S. B. Mandal N. P. Singh O. N. (2021). Genetic gain for rice yield in rainfed environments in India. Field Crop Res. 260, 107977. 10.1016/j.fcr.2020.107977 PMC7722510 33390645

[B29] Lenth R. Singmann H. Love J. Buerkner P. Herve M. (2019). Estimated marginal means, aka least-squares means. R package version 1.3.2. Available at: https://www.rdocumentation.org/packages/emmeans/versions/1.3.2/topics/emmeans-package .

[B30] Lorenz A. J. Chao S. Asoro F. G. Heffner E. L. Hayashi T. Iwata H. (2011). Genomic selection in plant breeding: knowledge and prospects. Adv. Agron. 110, 77–123. Elsevier. 10.1016/B978-0-12-385531-2.00002-5

[B31] Lorenz A. J. Smith K. P. Jannink J. L. (2012). Potential and optimization of genomic selection for Fusarium head blight resistance in six-row barley. Crop Sci. 52, 1609–1621. 10.2135/cropsci2011.09.0503

[B32] Lush J. L. (1937). Animal breeding plans. Ames: Iowa State College Press.

[B33] Meuwissen T. H. E. Hayes B. J. Goddard M. E. (2001). Prediction of total genetic value using genome-wide dense marker maps. Genetics 157, 1819–1829. 10.1093/genetics/157.4.1819 11290733 PMC1461589

[B34] Onogi A. Ideta O. Inoshita Y. Ebana K. Yoshioka T. Yamasaki M. (2015). Exploring the areas of applicability of whole-genome prediction methods for Asian rice (*Oryza sativa* L.) Theor. Appl. Genet. 128, 41–53. 10.1007/s00122-014-2411-y 25341369

[B35] Onogi A. Watanabe M. Mochizuki T. Hayashi T. Nakagawa H. Hasegawa T. (2016). Toward integration of genomic selection with crop modelling: the development of an integrated approach to predicting rice heading dates. Theor. Appl. Genet. 129, 805–817. 10.1007/s00122-016-2667-5 26791836

[B36] Park T. Casella G. (2008). The bayesian LASSO. J. Am. Stat. Assoc. 103, 681–686. 10.1198/016214508000000337

[B37] Piepho H. P. Möhring J. Melchinger A. E. Büchse A. (2008). BLUP for phenotypic selection in plant breeding and variety testing. Euphytica 161, 209–228. 10.1007/s10681-007-9449-8

[B38] Piepho H. P. Möhring J. Pfugfelder M. Hermann W. Williams E. (2015). Problems in parameter estimation for power and AR(1) models of spatial correlation in designed field experiments. Commun. Biom. Crop Sci. 10, 3–16.

[B39] Rahman M. A. Quddus M. R. Jahan N. Rahman M. A. Sarker M. R. A. Hossain H. (2019). Field rapid generation advance: An effective technique for industrial scale rice breeding program. The Experiment 47, 2659–2670.

[B40] Rahman M. N. F. Malik W. A. Kabir M. S. Baten M. A. Hossain M. I. Paul D. N. R. (2023). 50 years of rice breeding in Bangladesh: Genetic yield trends. Theor. Appl. Genet. 126, 1–13. 10.1007/s00122-023-04260-x PMC9867671 36680594

[B41] Resende M. F. Jr. Munoz P. Acosta J. J. Peter G. F. Davis J. M. Grattapaglia D. (2012). Accelerating the domestication of trees using genomic selection: accuracy of prediction models across ages and environments. New Phytol. 193, 617–624. 10.1111/j.1469-8137.2011.03895.x 21973055

[B42] Rikkerink E. H. Oraguzie N. C. Gardiner S. E. (2007). “Prospects of association mapping in perennial horticultural crops,” in Association mapping in plants. Editors Oraguzie N. C. Rikkerink E. H. A. Gardiner S. E. De Silva H. N. (New York: Springer), 249–269. 10.1007/978-0-387-36011-9_11

[B43] Rutkoski J. E. Heffner E. L. Sorrells M. E. (2011). Genomic selection for durable stem rust resistance in wheat. Euphytica 179, 161–173. 10.1007/s10681-010-0301-1

[B44] Rutkoski J. Benson J. Jia Y. Brown-Guedira G. Jannink J. L. Sorrells M. (2012). Evaluation of genomic prediction methods for Fusarium head blight resistance in wheat. Plant Genome 5, 51–61. 10.3835/plantgenome2012.02.0001

[B45] Salam M. Kabir M. S. Islam A. Sarkar M. A. R. Mamun M. Rahman M. (2020). Doubling rice productivity in Bangladesh: A way to achieving SDG 2 and moving forward. Bangladesh Rice J. 24, 1–47. 10.3329/brj.v24i2.53447

[B46] Sallam A. H. Endelman J. B. Jannink J. L. Smith K. P. (2015). Assessing genomic selection prediction accuracy in a dynamic barley breeding population. Plant Genome 8, eplantgenome2014.05.0020. 10.3835/plantgenome2014.05.0020 33228279

[B47] Schaeffer L. R. (2006). Strategy for applying genome-wide selection in dairy cattle. J. Anim. Breed. Genet. 123, 218–223. 10.1111/j.1439-0388.2006.00595.x 16882088

[B48] Shanmugavel P. Ramasamy G. Vellingiri G. Marimuthu R. Thiyagarajan K. (2022). “Speed breeding: A propitious technique for accelerated crop improvement,” in Plant breeding - new perspectives (IntechOpen). 10.5772/intechopen.105533

[B49] Smith A. B. Cullis B. R. (2018). Plant breeding selection tools built on factor analytic mixed models for multi-environment trial data. Euphytica 214, 143. 10.1007/s10681-018-2220-5

[B50] Spilke J. Richter C. Piepho H. P. (2010). Model selection and its consequences for different split-plot designs with spatial covariance and trend. Plant Breed. 129, 590–598. 10.1111/j.1439-0523.2010.01795.x

[B51] Spindel J. Iwata H. (2018). “Genomic selection in rice breeding,” in Rice genomics, genetics and breeding (Springer), 473–496. 10.1007/978-981-10-7461-5_24

[B52] Spindel J. Begum H. Akdemir D. Virk P. Collard B. Redona E. (2015). Genomic selection and association mapping in rice (oryza sativa): effect of trait genetic architecture, training population composition, marker number and statistical model on accuracy of rice genomic selection in elite, tropical rice breeding lines. PLoS Genet. 11, e1004982. 10.1371/journal.pgen.1004982 25689273 PMC4334555

[B53] Spindel J. Begum H. Akdemir D. Collard B. Redoña E. Jannink J. (2016). Genome-wide prediction models that incorporate de novo GWAS are a powerful new tool for tropical rice improvement. Heredity 116, 395–408. 10.1038/hdy.2015.113 26860200 PMC4806696

[B54] Venot E. Barbat A. Boichard D. Ducrocq V. Croiseau P. Frit S. (2016). “French genomic experience: genomics for all ruminant species,” in Proceedings of the 2016 Interbull Meeting, Puerto Varas (Chili).

[B55] Wang Y. Wang D. Deng X. Liu J. Sun P. Liu Y. (2012). Molecular mapping of the blast resistance genes Pi2-1 and Pi51 (t) in the durably resistant rice ‘Tianjingyeshengdao. Phytopathology 102, 779–786. 10.1094/PHYTO-03-12-0042-R 22779744

[B56] Wang X. Li L. Yang Z. Zheng X. Yu S. Xu C. (2017). Predicting rice hybrid performance using univariate and multivariate GBLUP models based on North Carolina mating design II. Heredity 118, 302–310. 10.1038/hdy.2016.87 27649618 PMC5315526

[B57] Whittaker J. C. Thompson R. Denham M. C. (2000). Marker-assisted selection using ridge regression. Genet. Res. 75, 249–252. 10.1017/S0016672399004462 10816982

[B58] Wiggans G. R. Cole J. B. Hubbard S. M. Sonstegard T. S. (2017). Genomic selection in dairy cattle: the USDA experience. Annu. Rev. Anim. Biosci. 5, 309–327. 10.1146/annurev-animal-021815-111422 27860491

[B59] Xu Y. Crouch J. H. (2008). Marker-assisted selection in plant breeding: from publications to practice. Crop Sci. 48, 391–407. 10.2135/cropsci2007.04.0191

[B60] Xu S. Zhu D. Zhang Q. (2014). Predicting hybrid performance in rice using genomic best linear unbiased prediction. Proc. Natl. Acad. Sci. U. S. A. 111, 12456–12461. 10.1073/pnas.1413750111 25114224 PMC4151732

[B61] Xu Y. Ma K. Zhao Y. Wang X. Zhou K. Yu G. (2021). Genomic selection: A breakthrough technology in rice breeding. Crop J. 9, 669–677. 10.1016/j.cj.2021.03.008

