# Supplementary material for: Enhancing genetic gain through the application of genomic selection in developing irrigated rice for the favorable ecosystem in Bangladesh
Source: Front Genet. 2023 Feb 22;14:1083221. doi: 10.3389/fgene.2023.1083221 (PMC9992429; doi:10.3389/fgene.2023.1083221)
Supplement: Supplementary file 1 [file Table1.docx]

**Supplementary Table S1** Meta data and descriptive statistics of the analysis of the breeding lines for yield during Boro season of 2018-19 to 2021-22 under favorable ecosystems

|  | Year of field trial | | | |
| --- | --- | --- | --- | --- |
|  | 2018-19 | 2019-20 | 2020-21 | 2021-22 |
| No. of genotypes tested | 431 | 816 | 1491 | 1029 |
| No of trial | 26 | 43 | 71 | 43 |
| No. of location | 1 | 9 | 1 | 7 |
| Trial design | RCB, SA | RCB | RCB | RCB, ARCB |
| Range (t ha^-1^) | 5.31-6.32 | 4.52 – 6.32 | 5.05– 6.15 | 5.11 – 6.56 |
| Average (t ha^-1^) | 5.77±0.026 | 5.81±0.14 | 5.79±0.20 | 5.77±0.27 |
| CV (%) | 4.44 | 2.45 | 3.46 | 4.65 |
