# Supplementary figures and images for: Enhancing genetic gain through the application of genomic selection in developing irrigated rice for the favorable ecosystem in Bangladesh
Source: Front Genet. 2023 Feb 22;14:1083221. doi: 10.3389/fgene.2023.1083221 (PMC9992429; doi:10.3389/fgene.2023.1083221)

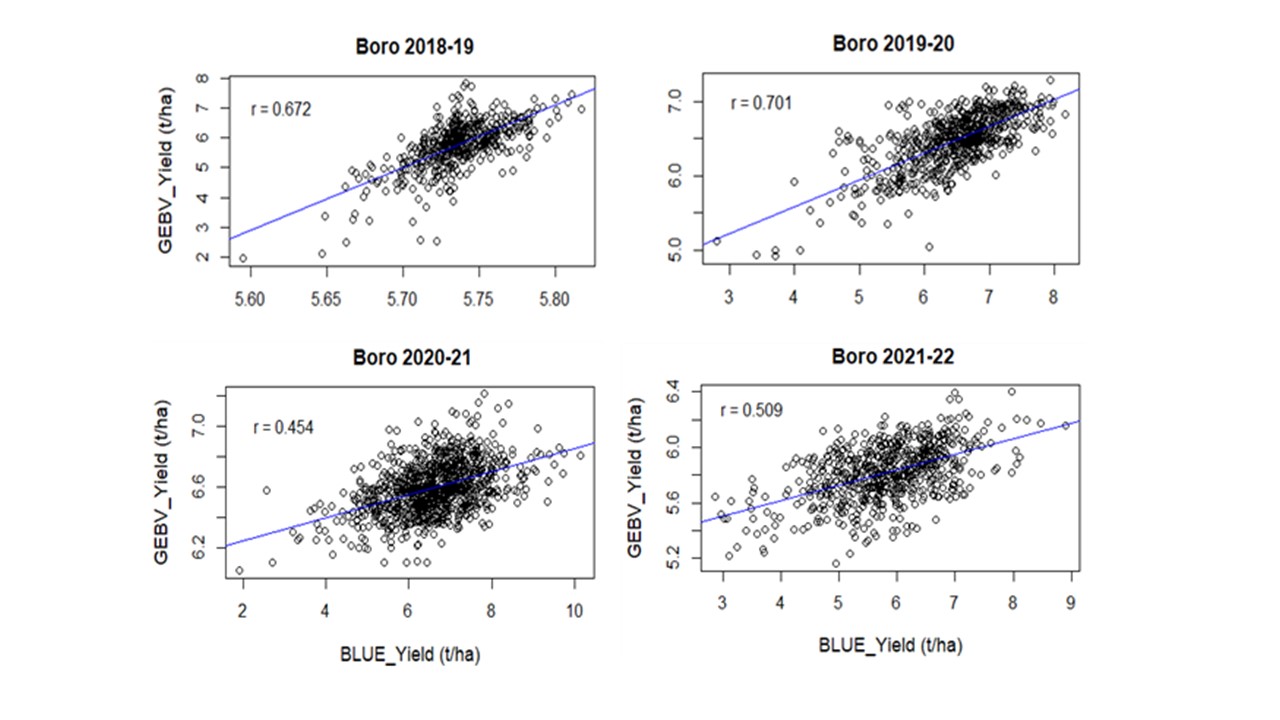

Supplement: Supplementary file 2 [file Image1.JPEG]
