# Supplementary material for: Enhancing genetic gain through the application of genomic selection in developing irrigated rice for the favorable ecosystem in Bangladesh
Source: Front Genet. 2023 Feb 22;14:1083221. doi: 10.3389/fgene.2023.1083221 (PMC9992429; doi:10.3389/fgene.2023.1083221)
Supplement: Supplementary file 3 [file Table2.docx]

**Supplementary Table S2** Contribution of two mega varieties BRRI dhan28 and BRRI dhan29 and landrace varieties in 1428 crosses made from 1994 to 2022 for the development of irrigated rice

| Variety | No of crosses that involved mega varieties | Proportion (%) |
| --- | --- | --- |
| BRRI dhan28 | 194 | 13.58 |
| BRRI dhan29 | 216 | 15.12 |
| Landrace Variety | 556 | 38.94 |
