# Supplementary material for: Enhancing genetic gain through the application of genomic selection in developing irrigated rice for the favorable ecosystem in Bangladesh
Source: Front Genet. 2023 Feb 22;14:1083221. doi: 10.3389/fgene.2023.1083221 (PMC9992429; doi:10.3389/fgene.2023.1083221)
Supplement: Supplementary file 4 [file Table3.docx]

**Supplementary Table S3** Timeframe of the current breeding cycle of irrigated breeding program

| Breeding stage | Years Required | Crop season |
| --- | --- | --- |
| Hybridization | 1.0 | T.Aman +Boro |
| F2-F5 in RGA | 1.5 | T.Aman +Boro |
| F5 in LST | 0.5 | T.Aman |
| OYT | 0.5 | Boro |
| Phenotyping for Key target traits | 0.5 | T.Aman |
| AYT | 0.5 | Boro |
| Total | 4.5 |  |
